# Biomimetic separations in chemistry and life sciences

**DOI:** 10.1007/s00604-025-06980-x

**Published:** 2025-02-04

**Authors:** Fotios Tsopelas, Chrysanthos Stergiopoulos, Panagiotis Danias, Anna Tsantili-Kakoulidou

**Affiliations:** 1 Laboratory of Inorganic and Analytical Chemistry, School of Chemical Engineering, National Technical University of Athens, Iroon Polytechniou 9, 15780 Zografou Athens, Greece; 2 Department of Pharmaceutical Chemistry, School of Pharmacy, National and Kapodistrian University of Athens, Panepistimiopolis, 15771 Zografou Athens, Greece

**Keywords:** Biomimetics, Biomimetic affinity chromatography, Immobilized artificial membrane (IAM) chromatography, Protein purification, Drug design

## Abstract

Since Otto Schmitt introduced the term “biomimetics” in 1957, the imitation of biological systems to develop separation methods and simulate biological processes has seen continuous growth, particularly over the past five decades. The biomimetic approach relies on the use of specific ligands—biospecific, biomimetic, or synthetic—which target biomolecules, such as proteins, antibodies, nucleic acids, enzymes, drugs, pesticides, and other bioactive analytes. This review highlights advances in biomimetic separations, focusing on biomimetic liquid chromatography (including immobilized artificial membrane chromatography, cell membrane chromatography, biomimetic affinity chromatography, weak affinity chromatography, micellar liquid chromatography, immobilized liposome chromatography, and liposome electrokinetic capillary chromatography) for the complex separation and purification of biomolecules and other important chemical compounds. It also explores their application in studying drug-receptor interactions, screening chemical permeability, absorption, distribution, toxicity, as well as predicting environmental risks. Additionally, this review discusses the application of biomimetic magnetic nanoparticles, which leverage biological membranes and proteins for drug discovery, protein purification, and diagnostics.

**Graphical Abstract:**

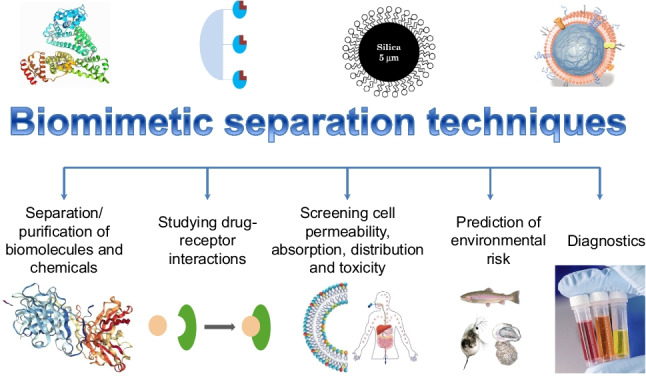

## The concept of “biomimetics”

**Fig. 1:**
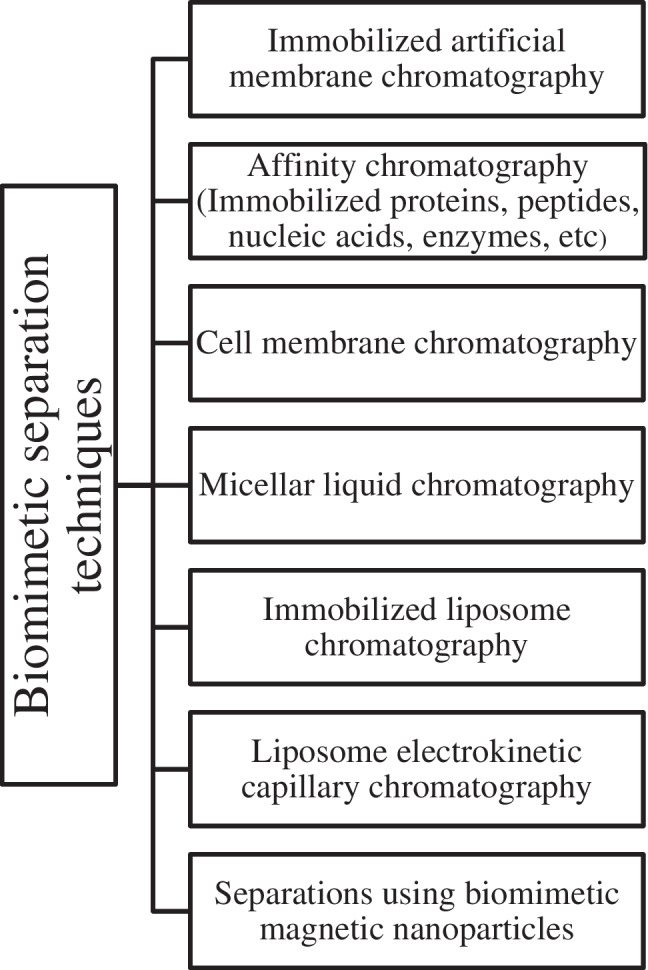
Biomimetic separation approaches

Biomimetics, an interdisciplinary field encompassing natural sciences, engineering, and materials sciences, involves emulating biological systems, mechanisms, and processes, to develop materials, devices, and processes [1] for the purpose of addressing complex challenges. Its origins trace back to humanity’s observation and imitation of solutions found in nature, leading to advancements such as flying machines and powered airplanes in the early twentieth century [2]. The term “biomimetics,” derived from the Greek word “bios” (life) and the suffix “imimetic” (mimesis/imitation) [3] was formally introduced by Otto Schmitt in 1957 during his doctoral studies, and subsequently, in 1960, Jack E. Steele coined the term “bionically” [4]. The concept gained traction, with the term “biomimetic” being defined in a 1969 paper and later incorporated into the dictionary in 1974. According to the ISO Standard 18,458:2015 [5], biomimetics is defined as the “interdisciplinary cooperation of biology and technology or other fields of innovation to solve practical problems through the function analysis of biological systems, their abstraction into models, and the transfer into and application of these models to the solution.” It is worth mentioning that transferring an idea or mechanism from living systems to non-living ones is a complex task. Simply copying a biological prototype may not succeed, even if modern technology makes it feasible. Presently, biomimetics endeavors to harness technology to mimic nature, aiming to improve human life and enhance quality of life [6]. Timely applications of biomimetics involve targeted drug delivery systems, medical devices, tissue engineering and regenerative medicine as well as robotics. In chemistry, biomimetics have been applied in fields, such as organic chemistry (organic synthesis that mimics biological synthesis processed in living organisms), biochemistry (e.g., purification of proteins), analytical chemistry (e.g., analytical separations), and medicinal chemistry (e.g., assessment of biological properties of bioactive species) [7–9]. The present review aims to cover the three main categories related to biomimetic separations, which are presented in Fig. 1 and in fact mimic the processes between lipophilic and aqueous phases within a biological or eco-system. It is focused on advances in biomimetic liquid chromatography, in liposome electrokinetic capillary chromatography, and in biomimetic magnetic nanomaterials and the fields that these technologies are applied. In the core of the above technologies is the use of natural (native), genetically modified, or artificial phospholipid membranes, as well as proteins to prepare biomimetic stationary phases or biomimetic nanoparticles (BNP). After an overview of the principles and underlying mechanism of each technology, their recent applications are discussed, highlighting their role in protein separation and purification, chiral separations and diagnostics, while their increasing impact in early drug discovery and in environmental sciences are emphasized.

## Biomimetic chromatography

Biomimetic chromatography encompasses immobilized artificial membrane chromatography, affinity chromatography, and cell membrane chromatography, distinguished by the type of the biological agent incorporated in the stationary phase. Micellar chromatography may be considered to belong in the same family of chromatographic techniques due to its ability to simulate biological environments. In this case, simulation of the biological environment is achieved by the formation of micelles in the mobile phase upon addition of different surfactants, mimicking the amphiphilic nature of biological membranes.

### Immobilized artificial membrane chromatography

Immobilized artificial membrane chromatography constitutes a prominent type of biomimetic chromatography utilizing stationary phases comprised of immobilized phospholipids, predominantly phosphatidylcholine on a silica support. It combines the simulation of the fluid environment of cell membranes with rapid chromatographic measurements [10–14]. Cell membranes constitute the environment for several types of molecular processes and membrane mimetics requires surfaces that mimic the physicochemical environment of biological membranes [15, 16]. In a biological membrane, double chain phospholipids are organized into a bilayer. The latter serves as the framework to embed all other components of the membrane. The technology of chromatographic columns succeed in the preparation of a range of artificial membranes, and their immobilization to appropriate solid support material (e.g., silica). These artificial membranes possess similarities to biological membranes and they mimic their amphiphilic microenvironment [10, 11, 13–18].

The first silica-based immobilized artificial membrane (IAM) column, which was referred to as IAM.PC, was prepared by covalently linking phosphatidylcholine (PC) analogue to silica-propylamine via their ω-carboxylic group on the C2 fatty acid chain by Charles Pidgeon in 1989 [15]. The research focused on the immobilization of phosphatidylcholine (PC) analogues; however, the group of C. Pidgeon succeeded in the immobilization of analogues of phosphatidylglycerol (PG), phosphatidylethanolamine (PE), phosphatidylserine (PS), and phosphatidic acid (PA) [19]. The first generation of IAM columns, namely IAM.PC.DD contained single-chain phosphatidylcholine and lacks the glycerol moiety of natural phospholipids [10, 17, 18]. However, double chain PC stationary phases (e.g., IAM.PC.MG) may provide a better insight into biological partitioning processes of compounds [18, 20]. The commercially available double chain IAM.PC.DD2 and IAM.PC.MG columns differ in the end-capping of the free prolylamino residues. More to the point, end-capping is performed by using C10 and C3 acyl groups for IAM.PC.DD2 and methylglycolate for IAM.PC.MG stationary phase [17]. However, studies using large data sets of structurally diverse drugs underscored small differences in retention on the two IAM columns [18].

IAM retention is primarily governed by partitioning; however, it is affected by electrostatic interactions, which are more pronounced between protonated bases and phosphate anions, located close to the hydrophobic core of the prospholipids [10, 11, 18, 21, 22]. The positively charged choline nitrogen is located at the outer extreme of the IAM surface and, therefore, is less accessible to interactions with anions of acidic compounds [10, 18].

IAM stationary phases are usually employed using buffers and phosphate-buffered saline (PBS) is preferred to enhance biomimetic simulation. Ammonium acetate buffer is recommended due to its compatibility with mass spectrometry, which enhance throughput, compared to traditional UV detection methods, though with some differences in the elution order of analytes [23]. Although, IAM stationary phases can be employed with pure aqueous phase, an organic modifier can be added to facilitate elution of lipophilic compounds. Acetonitrile is the organic additive of choice. Methanol and ethanol should be avoided as organic phase additives because they can provoke hydrocarbon leaching from IAM stationary phases containing phosphatidylcholine [24].

In fact, IAM chromatography finds limited application in separations in the analytical sense of the term. IAM.PC. phases have been employed in simplified protein isolation and purification, allowing for rapid purification of membrane proteins while maintaining their biological activity.

In early studies, Pidgeon et al. successfully demonstrated the potential of home-made IAM stationary phases for the separation and purification of peptides (containing cysteine or not) [15], cholesterol binding protein [25], membrane proteins, such as cytochrome P450 proteins [16], *N*-acylphosphatidylethanolamine synthetase [26], rat liver aldolase [27], bovine pancreatic phospholipase A2 (PLA 2) [19]. It should be noted that protein fractionation and purification is a challenging task in separation science, especially crucial in the post-genome era, where proteomics plays a vital role in understanding diseases beyond genetic changes. Efficient and selective methods are necessary for isolating proteins from biological samples that contain nucleic acids, carbohydrates, and other substances [28, 29]. Purification processes should ensure proteins are free of contaminants and isoforms, essential for pharmaceutical applications and clinical research [30]. However, there is a lack of applications in analytical scale for the separation of proteins due to the low efficiency of commercial IAM stationary phases. The use of IAM stationary phases may be extended to the purification of viral membrane proteins for multivalent vaccines and removal of endotoxins from pharmaceuticals [15].

On the other hand, retention on IAM stationary phases have been proved as a valuable tool to screen chemicals regarding their potential to cross or bind to biological membranes allowing estimation of crucial pharmacokinetic properties such as absorption and distribution, as well as toxicity of candidate drugs. They have also significant applications to the ecotoxicological risk assessment of chemicals (e.g., pesticides) as a prerequisite to enter the market [31, 32]. IAM-based quantitative retention-activity relationships (QRARs) or quantitative retention-property relationships (QRPRs) [33, 34] use the logarithm of retention factor, or the chromatographic hydrophobicity index CHI-IAM to model the target property.

The logarithm of retention factor logk is defined by the formula (1):

1 $$logk=\text{log}\left(\frac{{t}_{r}-{t}_{0}}{{t}_{0}}\right)$$

where t_r_ is the retention time of the compound under investigation and t_0_ is the column void time. In the case of IAM stationary phases, l-cystine, KIO_3_ and sodium citrate are good choices to be used as void time markers. Suggestions for the selection of the appropriate void time marker according to the chromatographic column and acidic or neutral mobile phases are given in ref. [35].

For the construction of QRARs and QRPRs, logk_w_ usually is considered, where the subscript w denotes that measurements are performed in 100% aqueous mobile phase (actual logk_w_ values). For lipophilic drugs, logk_w_ values are obtained by linear extrapolation of isocratic logk values measured in presence of (at least three) different percentages of organic modifier (acetonitrile), according to Eq. (2):

2 $$logk=-S\cdot \varphi +log{k}_{w}$$

where S is the slope and logk_w_ is the intercept of the regression line.

The chromatographic hydrophobicity index CHI-IAM is defined in analogy with the CHI index in reversed phase HPLC [34]. CHI-IAM corresponds to the percentage of organic modifier (acetonitrile), which produces equal partitioning of the solute between the IAM stationary phase and the mobile phase, e.g., logk = 0. In this sense, CHI-IAM equals the quotient logk_w_/S of Eq. (2), and is designated also as φ_0_. Gradient retention times are calibrated against isocratically obtained ϕ_0_ values, labeled thereafter as CHI-IAM. As a next step the calibration plot is used for rapid determination of CHI_(IAM)_ of other compounds [34, 36, 37].

IAM retention factors are widely used in modeling permeability through various biological membranes such as the gastrointestinal [10, 11, 38], blood brain barrier [39, 40], or skin [41, 42] in most cases in combination with additional molecular descriptors, reflecting mainly bulk and/or polarity/hydrogen bonding potential. CHI-IAM upon its transformation to the thermodynamic constant logK_(IAM)_ [43, 44] can be employed for the estimation of composite pharmacokinetic properties such as volume of distribution, unbound volume of distribution, fraction unbound, in combination with retention measured on HSA chromatography, see “Biomimetic affinity chromatography” section. Standard models based on the weighted sum of retention on both biomimetic stationary phases are suggested, according to Eq. (3):

3 $$log(Property)=a\cdot log{K}_{(IAM)}+b\cdot {K}_{(HSA)}+c$$

In these cases, logK_IAM_ accounts for tissue binding with positive sign for volume of distribution and unbound volume of distribution and negative sign for fraction unbound [45]. Further application of IAM chromatography in combination with retention on an AGP column (see “Biomimetic affinity chromatography”) concerns the evaluation of drug candidates to inhibit the hERG (human Ether-a-go-go Related Gene) channel responsible for the potassium cation flux, which can cause cardiotoxicity [46]. In recent years, applications of IAM chromatography have been expanded to environmental sciences/ ecotoxicology to predict acute aquatic toxicity (i.e., lethal concentration of fish species and effective concentration of water flee/Daphnia Magna and Eastern Oyster) of pesticides [47] or drugs [48].

Apart from the phosphatidylcholine containing IAM stationary phases, other phospholipids have been immobilized or incorporated by a co-polymerization procedure on IAM stationary phases. A prototype for the preparation of sphingomyelin stationary phase has been reported [49]. Sphingomyelin is the most abundant complex sphingolipid in human cells. It is an amphoteric lipid composed of sphingosine, fatty acid, and phosphorylcholine [49]. Due to its abundance in brain, the sphingomyelin stationary phase has been utilized to model blood–brain barrier passage [49].

In the last decade, monolithic IAM stationary phases have been developed and proposed as alternatives. Monolith IAM columns can be prepared by in situ co-polymerization procedure using a long alkyl chain phosphatidylcholine as functional monomer, such as 12-methacryloyl dodecylphosphocholine (MDPC) and ethylene dimethacrylate (EDMA) as crosslinker [50, 51]. Advantages of monolithic IAM stationary phases include high stability across a wide pH range [51]. Monolithic IAM stationary phases have been used for the early screening of candidate drugs in terms of drug-membrane interactions [50] as well as for the evaluation of phospholipidosis risk [52], a lysosomal storage disorder characterized by the excess accumulation of phospholipids in tissues. A synopsis concerning the different types of IAM stationary phases used to model biological processes is presented in Table 1. A comprehensive presentation of IAM applications with a compilation of IAM based models can be found in ref. [13, 14].

**Table 1:** Different types of IAM stationary phases according to phospholipids immobilized, commercial availability and biological processes modeled

Type of IAM (Phospholipid immobilized)	Biological processes modeled	Commercial availability	Reference
Phosphatidylcholine (IAM.PC.DD2, IAM.PC.MG)	Permeability (human oral absorption), skin partition, volume of distribution, blood–brain barrier passage, cardiotoxicity, ecotoxicological endpoints	Regis Technologies Inc. (Morton Grove, IL, U.S.A.)	10, 11, 13, 14, 33, 34, 38, 41, 42, 43, 45–48
Phosphatidylserine	Drug-induced phospholipidosis	–	14, 19
Sphingomyelin	Passage of chemicals through blood–brain barrier	–	49
Mixed phosphatidylcholine and phosphatidic acid (monolithic)	Drug-induced phospholipidosis	–	14
Mixed phospholipid functionalized monolithic column containing n-dodecylphosphocholine and n-dodecylphosphoserine	Drug-induced phospholipidosis	–	52

Besides their use in the prediction of drug permeability and pharmacokinetic behavior, monolithic IAM stationary phases can also be employed for separation of various analytes. Zhao et al. postulated the separation of 4 proteins (bovine serum albumin, lysozyme from egg, cytochrome C and ribonuclease) as well as the separation of 11 basic drugs (caffeine, zidovudine, lidocaine, famotidine, phenacetin, indomethacin, metolazone, nitrendipine, astemizole, tamoxifen, sertraline) [50]. This demonstrates the versatility of monolithic IAM columns in both drug screening and analytical separations.

The unique advantages of IAM chromatography in purifying proteins and modeling biological processes provide valuable insights, particularly to the fields of drug discovery and ecotoxicology. However, its routine application is limited by the lack of commercially available relevant stationary phases [14]. As noted, only IAM.PC columns (IAM.PC.MG and IAM.PC.DD2) are commercially available, exclusively manufactured by Regis Technologies Inc. (Morton Grove, IL, U.S.A.). These columns are approximately ten times more expensive than traditional reversed-phase stationary phase and their specialized nature has led to limited adoption by laboratories.

### Cell membrane chromatography

Cell membrane chromatography (CMC) is a biological affinity chromatographic technique, where specific cell membranes containing certain receptors are used as stationary phases [53]. CMC stationary phases are not commercially available and a simple technique to prepare them is the immersion of silica into a suspension of cell membranes resulting in a coverage of the whole surface of silica by the cell membranes due to the irreversible adsorption of silanol groups (Si–OH) on the silica surface and the self-fusion of the cell membranes [53, 54]. CMC combines the characteristics of cell membranes with the user-friendly chromatographic separation and it can be used for chiral separations [55] as well as for the investigation of drug-receptor interactions [53, 56, 57]. In the latter case, the method, called also “receptor affinity chromatography,” the affinity of a drug with membrane receptors is usually studied through zonal elution [54] or frontal analysis [56, 57]. It is worth mentioning that zonal elution is performed using a mobile phase containing a fixed amount of a competitive agent, while in frontal analysis, the analyte in solution in a certain concentration is applied continuously in the stationary phase, resulting in a progressive saturation of the binding sites of the column and a subsequent increase of the analyte concentration reaching a plateau [58]. CMC proved a practical approach to screen active components from herbal medicines [54, 59–61]. The identification of active ingredients with affinity to cell membranes receptors can be achieved using a two-dimensional liquid chromatography approaches, involving a retention/separation in CMC column followed by UV detection and the (online or offline) analysis of retention fractions by LC–MS or GC–MS [54, 61]. This integrative approach allows for the precise identification and characterization of bioactive compounds, offering significant potential in pharmaceutical and medicinal research.

### Biomimetic affinity chromatography

Affinity chromatography constitutes a powerful and highly selective type of liquid chromatography based on molecular recognition. It uses a biologically related agent immobilized on the stationary phase to separate and/or purify specific molecules. The method leverages specific interactions between target molecules and affinity ligands, mimicking biomolecule substrates, facilitating the separation and isolation of biomolecules from complex samples [6, 62].

**Fig. 2:**
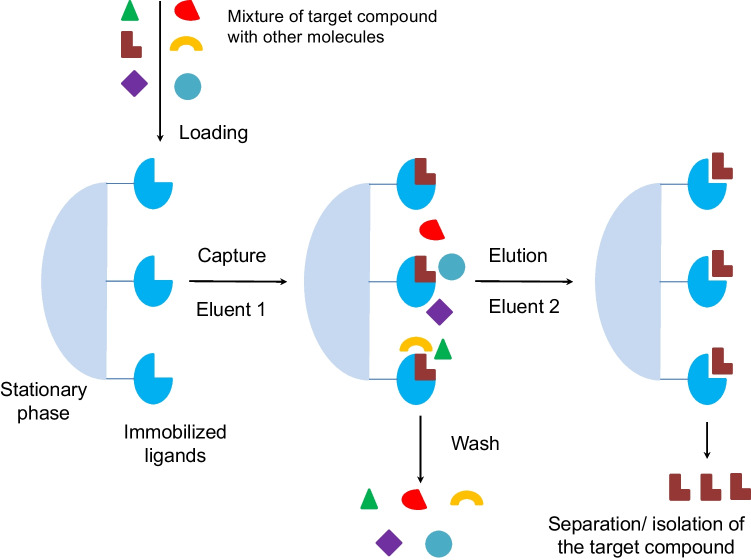
Flowchart of the affinity chromatography process

Initially developed by Cuatrecasas, Wilchek, and Anfinsen in 1968 to purify enzymes using their substrates and inhibitors as ligands [63], affinity chromatography has since evolved and is now applied in new fields, such as biosensing, drug delivery systems, and tissue engineering studies [6]. Figure 2 illustrates the flowchart of the affinity chromatography process, where a ligand is typically covalently immobilized onto a support material using a spacer arm [6, 64]. Nonspecific immobilization, mainly through physical adsorption is also possible [62]. After the mixture containing the target molecule is then loaded into the affinity column, the bound target molecule is eluted by adjusting parameters such as pH, ionic strength, and temperature.

Affinity ligands can be classified into biospecific ligands (e.g., antigen–antibody, lectin-glycoprotein), biomimetic ligands (e.g., peptides and triazine-based ligands), and synthetic ligands (e.g. dyes, metal-chelators) [6, 64]. Table 2 lists the primary ligands employed in biomimetic affinity chromatography along with their target compounds and applications. These ligands can either possess high-specificity, targeting only one or a few closely related molecules (e.g., antibodies for the binding of antigens, substrates or inhibitors for the separation of enzymes and single-strand nucleic acids for the retention of complementary sequences of DNA or RNA) or moderate specificity (general ligands), targeting a broader class of related molecules (e.g., lectins for carbohydrate-containing compounds, proteins A and G for immunoglobulins) [62].

**Table 2:** The most important ligands employed in biomimetic affinity chromatography

Type of ligand	Retained compounds	Comments	References
Immunoglobulin-binding proteins (Protein A, Protein G, Protein L)	Antibodies		69, 83–89
Antibodies	Antigens: Drugs and hormones, proteins, peptides, viruses, cell components	High cost, instability, leakage problems	4, 67, 68
(i) Inhibitors, substrates, cofactors (ii) Enzymes	(i) Enzymes (ii) Inhibitors, drugs (including chiral structures) and other entities		69–72
Lectins	Sugars, glycolipids and glycoproteins		69, 80–82
Nucleic acids	Complementary nucleic acids and DNA/RNA-binding proteins		4, 73–79
Serum proteins: Human Serum Albumin (HSA), Bovine Serum Albumin (BSA) and Alpha-1 acid glycoprotein (AGP)	Drugs, hormones, fatty acids, pesticides		4, 103–112
Small peptides and triazine-based ligands	Antibodies	Designed by techniques such as combinatorial chemistry, computer simulation	90–102
Lipids (artificial membranes, liposomes)	Drugs, hormones, fatty acids, pesticides, organic pollutants	Used in medicinal chemistry (drug design) for the investigation of molecules-membrane interactions and membrane permeability of chemicals	10–20, 25–34, 38–52, 116, 155–163

The advantage of biomimetic ligands lies in the fact that they are more affordable and stable, mimicking critical residues involved in recognition processes, whereas biospecific ligands can be costly and unstable, although they offer selectivity. Advanced technologies like computer-based screening and combinatorial methods help designing biomimetic ligands, meeting diverse purification needs and applications.

Retention on affinity chromatography is driven by hydrophobic and electrostatic interactions, Van der Waals forces as well as hydrogen bonding [65]. These interactions can create biospecificity, enabling the separation/purification of biomolecules, such as enzymes, antibodies, hormones, and nucleic acids. Non-biospecific affinity chromatography is predominantly conducted through metal affinity chromatography (MAC), which depends on the interaction between transition metals (e.g., nickel, zinc, iron and copper) and metal-binding sites on large molecules, primarily histidine residues [66]. As MAC is not truly biomimetic, its principles and applications are beyond the scope of this review.

Immunoaffinity chromatography is the most common type of biomimetic affinity chromatography [4]. It is based on the binding of an antibody to its specific antigen or target. Antigen–antibody interactions are strong and highly selective, covering a wide range of compounds [4, 67, 68]. The method has been effectively employed to isolate many compounds, including hormones and toxins to peptides, antibodies, enzymes, recombinant proteins and even viruses [4, 67, 68].

The understanding the roles and functions of enzymes, cofactors, substrates, and inhibitors has led to the immobilization of inhibitors, substrates, and cofactors onto HPLC stationary phases for the purpose of isolating enzymes [69, 70]. Immobilized enzymes such as lysozyme, pepsin, trypsin, α-chymotrypsin, and glycoamylases G1 and G2 have been employed to separate inhibitors, drugs and other solutes that bind to these enzymes [70–72].

Another type of biomimetic ligands for affinity chromatography is nucleic acids. The method is known as DNA affinity chromatography, which aims to retain and purify DNA-binding proteins. DNA affinity chromatography can be either specific or non-specific. In the specific approach, a particular DNA segment with a defined sequence, structure or restriction site is used as the affinity ligand to selectively interact specifically with the target protein. In the non-specific approach, a fragmented nuclear DNA (e.g., calf thymus DNA) is utilized to interact broadly with DNA-binding proteins [4]. Recent studies revealed that DNA affinity chromatography has been successful in isolating several DNA-binding proteins, including DNA and RNA polymerases, DNA repair proteins, helicases, histones, primases, restriction enzymes, teleomerases, topoisomerases, and transcription factors [73–75]. A related method is aptamer affinity chromatography [76]. Aptamers are synthetic oligonucleotide sequences generally based on DNA or RNA, which can be selected and optimized to bind specific molecules [4]. Aptamers are considered as alternatives to antibodies and are employed to bind a wide range of targets, ranging from small molecules (e.g. cocaine, diclofenac) to large entities, such as proteins and cells [76–79].

Lectin affinity chromatography uses lectins, which are non-immune and carbohydrate-binding proteins with high specificity for sugar-containing targets [69]. Concanavalin A is a commonly used lectin in this technique, specifically binding to high-mannose glycans and glycans with mannose branching [69]. Serial lectin affinity chromatography, where multiple lectin columns are combined, has been proposed to characterize glycans on the same glycoconjugate [80]. Monolith stationary phases have been developed using a combination of concanavalin A with wheat germ agglutinin [81] and microclumns containing concanavalin A or Aleuria Auranta lectin [82]. Lectins can also be used as affinity ligands in order to separate specific types of cells by interacting with cell surface agents, such as glycoproteins [4].

Affinity chromatography using immunoglobulin-binding proteins constitutes a crucial biomimetic separation tool, primarily for purifying antibodies. Antibodies are large Y-shaped proteins that recognize specific parts of foreign targets, known as antigens and are valuable in drug development and diagnostics [83]. The most significant binding agents in this process are proteins A and G. Protein A, derived from the bacterial cell wall of *Staphylococcus aureus*, is commonly employed for purifying various immunoglobulins subclasses [69, 84–86]. Protein A affinity resin, composed of five immunoglobulin-binding domains, possesses a high affinity for immunoglobulin G (IgG), specifically targeting the heavy chain of the Fc region with high specificity [87]. Proteins G and L, sourced from *Streptococcus* group G and *Peptostreptococcus magnus*, respectively offer different selectivity compared to Protein A and are also used as binding agents [4, 88, 89]. Mixtures of these proteins (e.g., A and G or G and L) have been effectively employed to separate and purify antibodies [4, 69, 84].

Protein A affinity chromatography has certain drawbacks, such as toxic ligand leakage, high cost, and harsh elution conditions [83, 90]. Peptide biomimetic affinity chromatography offers an alternative, leveraging highly specific interactions between short peptides (two to nine amino acids) and target proteins. These peptides can effectively compete with natural ligands for protein binding [91]. Selected based on the receptor’s 3D structure and active sites, linear or cyclic peptides are selected using tools like computer-aided screening and combinatorial chemistry [91]. The chosen peptide is subsequently coupled to a matrix using spacer arms, often with. polymer-grafted resins as a bridge between the matrix and ligand [83, 91, 92]. Wang et al. used flexible docking and molecular dynamics simulation to develop a new peptide resin (Ac-YFRH-4FF resin) for the purification of Human immunoglobulin G (hIgG) from the bovine albumin (BSA)-containing feedstock [90]. Yang et al. screened HWRGWV ligand from a synthetic solid-phase library and developed a resin capable of binding all human IgG subclasses and IgGs from various species, including bovine, mouse, goat, and rabbit and separating hIgG from mammalian cell culture medium [93–95]. Fang et al. developed a novel tetrapeptide ligand (Ac-FYHE) with high specificity for BSA, used for separating hIgG from BSA-containing feedstock and human serum and purifying monoclonal antibodies (mAbs) from CHO cell culture supernatant [83]. They also demonstrated that dextran can be well used as a spacer arm in peptide biomimetic chromatography to increase protein adsorption capacity under weak alkaline conditions, important for purifying proteins that cannot be separated under acidic and neutral conditions [92]. Kish et al. showed that cyclic peptides FSLLSH and FSLLHH, synthesized on Toyopearl chromatographic resins are effective for purifying recombinant human erythropoietin (rHUEPO) purification [96]. Ma et al. used six biomimetic affinity resins to fractionate *M*. *tuberculosis* cytoplasmic proteins, which were then analysed by liquid chromatography- mass spectrometry (LC–MS/MS), resulting in the detection of 1246 M. *tuberculosis* proteins [97]. Huang et al. synthesized a peptide affinity gel, SepFF-bPL, by coupling a biomimetic peptide ligand (bPL), FYWHCLDE onto Sepharose 6 Fast Flow (SepFF), which successfully captured targets from various feedstocks with high purity and yield, although it was less effective at removing host cell proteins and DNA [98]. Xu et al. developed a histidine-tagged cyclic peptide (HT25-cyclopeptide) functionalized monolithic material using the metal ion chelation, which was applied for the selective enrichment and purification of antibodies from cell culture media or human blood serum [99]. While peptide biomimetic affinity chromatography has some limitations, such as lower selectivity compared to protein A affinity chromatography [98] and moderate resin stability and reusability [96]. Yan et al. investigated the effect of the particle size of three matrix spheres with average particle sizes of 31, 60, and 85 μm (Matrix-31, Matrix-60, and Matrix-85, respectively) and they highlighted that small particle size resins can enhance the dynamic binding capacity and improve protein purification efficiency [100].

In addition to peptides, other biomimetic ligands with small molecular functional groups can also be used. These ligands are often designed using computer-aided molecular simulation, gene recombination, and combinatorial chemistry [101]. Ma et al. proposed an alkaline-tolerant biomimetic affinity resin (BiAC-A5-87) containing sulphonamide and ethylenediamine groups for the purification of Bovine Serum immunoglobulin (Bs-IgG), aimed at recycling the bovine blood from slaughter and converting it into high value bovine serum products [101]. Farzi-Khajeh et al. created biomimetic triazine-based affinity ligands, which were covalently attached to resins for fast and low-cost purification of immunoglobulin G from human and rabbit plasma [102].

Another important class of binding agents for biomimetic affinity chromatography is serum proteins, particularly human serum albumin (HSA), bovine serum albumin (BSA), and alpha-1 acid glycoprotein (AGP). HSA, the most abundant human plasma protein, primarily binds acidic compounds and possesses two main stereoselective drug-binding sites, known as Sudlow’s sites 1 and 2 [12]. BSA, the most abundant protein in bovine serum, has about six fatty acid- binding sites and two drug-binding sites, binding fatty acids, drugs, hormones, and aminoacids [4, 103]. AGP constitutes the second most important plasma protein, with concentrations that vary based on factors, such as disease, age, gender, age, and pregnancy. It mainly binds basic and neutral compounds [12]. HSA, BSA, and AGP have been employed as chiral stationary phases and various conditions have been studies to optimize chiral separations [4, 104–106]. Chiral separations are particularly challenging in Medicinal Chemistry due to the need to distinguish between stereoisomers-molecules with the same chemical structure but different spatial orientations. Enantiomers, a type of stereoisomers, are mirror images that can have different effects in biological systems, affecting absorption, distribution, metabolism, excretion, and molecular action [107, 108]. Therefore, enantioseparation and determination of optical purity are critical in drug development [109]. Beyond pharmaceuticals, chiral separations are also important in to agrochemicals and food additives as well [109]. These separations can also be achieved using molecularly imprinted polymers, carbohydrate phases based on amylose, cellulose, cyclodextrins, and cyclofructans as well as using enzymes and antibodies [4].

Apart from their use in chiral separations, human serum proteins can be employed for the investigation of molecule-protein interactions, a crucial issue especially in early drug discovery. Plasma proteins upon binding to drugs affect several biological processes, including the transport, distribution, metabolism, and excretion of chemical compounds, controlling the duration of their presence in the body, while they temporarily inactivate them since they cannot pass membranes to reach their targets.

Retention factors on plasma proteins stationary phases reflect both specific and non-specific interactions [12]. Strong retention on plasma protein stationary phases, particularly HSA columns, indicates strong protein binding in plasma [34, 110]. Τhe percentage of drug plasma protein binding (% PPB) can be estimated using retention factors, k, measured on HSA stationary phases according to the formula [34]:

4 $$\%PPB=100\frac{k}{k+1}$$

The Eq. (4) implies that there is a nonlinear relationship between retention factors and %PPB and a saturation curve is evident. For drugs with over 90% binding, minor differences in %PPB (e.g., 92% and 93%) can lead to significant variations in retention time [12]. Strong binding on HSA columns reduces the apparent volume of distribution, as drugs are retained in plasma bound to its proteins. The group of Valko et al. proposed a model for estimating apparent volume of distribution and fraction unbound in tissues based on retention on IAM in combination with HSA chromatography, as already mentioned in paragraph 2.1. [34, 43]. For this purpose, simple two-parameter relationships have been established, in the aim to serve as standard models [45].

The use of AGP column for estimating drug binding remains less explored [12, 111]. Vuignier et al. classified compounds as weak, medium, or strong binders to AGP using the logarithm of gradient retention factor [112]. To ensure that binding data are comparable across different laboratories, calibrating the chromatographic system is recommended. This calibration involves measuring data for a set of compounds with binding information available from other methodologies, such as equilibrium dialysis, ultrafiltration, and frontal analysis. Such calibration is crucial for achieving consistent data comparability between laboratories and improving reproducibility [33, 34].

Vallianatou et al. integrated HSA and AGP with IAM retention factors and molecular descriptors to construct hybrid models predicting drug brain disposition (e.g., brain permeability, ratio of unbound drug concentration in the brain interstitial fluid to the corresponding plasma concentration, unbound fraction in the brain, brain unbound volume of distribution) [40]. Additionally, HSA and AGP can be used to study drug affinity, binding sites, allosteric interactions, and the effects of protein modifications on drug binding [72, 113–115], which can provide insights into drug-drug interactions, such as competition for the same binding site [12]. Recently, retention on the AGP stationary phase was found to correlate with the lethal concentration 50 (LC_50_) of fish and the effective concentration (immobilization) (EC_50_) of water flea (*Daphnia magna* spp.), suggesting its potential as an ecotoxicological risk assessment tool [46].

The use of lipids as biological agents in bioaffinity chromatography (e.g., phospholipids in IAM chromatography, liposomes in immobilized liposome chromatography) is described in paragraphs 2.1 and 2.6. It should be noted that although affinity chromatography is an effective technique for separation and purification of important molecules (e.g., proteins, antibodies, enzymes, drugs), it possesses several notable drawbacks, particularly regarding cost and stability [116]. Only a limited number of relevant stationary phases are commercially available and they are relatively expensive, costing approximately ten times more than conventional reversed-phase columns. The process of coupling ligands to the stationary phase may necessitate the use of expensive and potentially hazardous chemicals. Additionally, ligand immobilization can vary among batches, leading to problems in reproducibility. Other problems include operation under restricted conditions (e.g., a narrow pH range), the short lifespan of stationary phases and destabilization during column regeneration, resulting in ligand leaching. For instance, the leaching of Protein A ligands during the elution phase may lead to their co-elution with the target compound [66].

### Weak affinity chromatography

A special case of affinity chromatography is weak affinity chromatography (WAC), which also uses proteins in the stationary phases but focuses on identifying specific interactions of ligands with the target protein. Indeed, WAC can analyze weak or transient biological interactions, such as binding strength and kinetics, while also providing separation and purification of the analyte being studied. When combined with mass spectrometry (MS) as the detector, the WAC-MS system becomes an effective technique for identifying the analyzed substances if appropriate standards are available [117]. However, in complex matrices and in the absence of standards, information provided by MS often needs to be combined with those provided from other analytical tools for the complete identification of the analyzed species.

WAC is mostly used in fragment screening, an approach in drug discovery [118], employing small molecules (≤ 300 MW) [119] that bind weakly to protein targets. Originating from Jencks’ additivity perception of binding energies [120], fragment-based drug discovery leverages the additive nature of binding energies and the multiplicative nature of affinities of linked fragments [121]. This method results in high ligand efficiency, with fewer heavy atoms and lower lipophilicity, and minimizes steric hindrance. The smaller fragment libraries (~ 1000 compounds) efficiently cover the chemical space compared to traditional libraries [122]. The first FDA-approved FBDD drug was Vemurafenib in 2011 [123], followed by Venetoclax in 2016 [124]. Many other FBDD-developed drugs, particularly kinase inhibitors, are in clinical trials.

Stationary phases of WAC are not commercially available, thus limiting its use. The in-house immobilization of the target protein can be performed either in situ or in batch. In situ immobilization is performed in standard columns (30 × 2.1 mm) containing porous silanized diol-based spherical silica particles. This involves oxidation of diol-silica to aldehyde silica and covalently coupling it to primary amine groups of the protein to form a Schiff base. Batch immobilization is recommended for large proteins or lipodisks onto silica particles as mixing of all components during reactions is necessary [125].

Following the principles of liquid chromatography, retention of analytes on WAC stationary phases depends on their binding with the target protein immobilized on the stationary phase, involving specific interactions with the active site of a target, while non-specific interactions may occur outside the active site of the target. WAC is sufficiently sensitive to detect weak interactions involved in fragment affinity and to distinguish specific interaction between ligand and target protein from undesired promiscuous binding. WAC allows the determination of association constant (dissociation constant) representing the strength of binding between ligand and a target protein and binding kinetics (association rate constant) by measuring the chromatographic peak. Frontal analysis allows the determination of the amount of active protein immobilized in the column [125].

Successful fragment screening applications on several soluble protein targets include proteases (a-thrombin, trypsin) [126–128], kinases (JAK) [129, 130], chaperones (heat shock protein 90 Hsp90) [131, 132] isomerases (peptidyl-propyl *cis*–trans isomerase NIMA interacting 1, PiN1), a protein–protein interaction (PPI) target [133]. The ability of WAC to separate and selectively determine affinities of stereoisomers and diastereoisomers during fragment screening was demonstrated in the case of the cyclin G-associated kinase and the a-thrombin target [127, 131].

The drawbacks of WAC include, among others, a labor-intensive and costly process for preparing the stationary phase, a short column lifespan, limited specificity due to potential non-specific binding, time-consuming optimization of chromatographic conditions (e.g., mobile phase composition and pH) and difficulty in eluting strongly retained compounds [125].

A promising alternative to protein stationary phases is the use of lipodisks, sterically stabilized bilayer disks used as model membranes in drug studies. They are planar and circular in shape, with polyethylene glycol (PEG) lipids located at their rim, providing steric protection against fusion and self-closure. Lipodisks avoid the polydispersity issues of liposomes and have surfaces that are more accessible to the surroundings. They can be immobilized on materials like Superdex gel beads to create columns for immobilized liposome chromatography (ILC). The first use of “proteolipodisks” for fragment screening of human aquaporin-1 (AQP1) in 2016 [134] marked significant progress, enabling WAC to include membrane proteins. Lipodisks facilitate the reconstitution of integral membrane proteins, making them suitable for drug permeability and interaction studies [125]. However, further investigation is needed especially in the area of immobilizing membrane proteins on lipodisks.

### Micellar liquid chromatography

Another case of biomimetic chromatography that has attracted considerable interest is micellar liquid chromatography (MLC). Unlike IAM and biomimetic affinity chromatography, MLC does not require a specific stationary phase. Instead, it uses a conventional inexpensive reversed-phase stationary phase, usually available in any analytical laboratory and a mobile phase containing a surfactant at a concentration above its critical micellar concentration (CMC) [135]. Under these conditions, the mobile phase contains both surfactant monomers and aggregates of generally 20 to 100 monomers, known as micelles. Surfactant monomers are absorbed onto the surface of the stationary phase via hydrophobic and silanophilic interactions [136], while the aggregates play the role of lipophilic droplets, which facilitates the elution of hydrophobic drugs. The adsorption of surfactant monomers leads to an ordered array of hydrocarbon chains and polar regions on the stationary phase surface, which thereupon resembles membranes providing a simulation of a biological environment. Thus, in the case of MLC, the biomimetic character is achieved by the interaction of stationary phase with the surfactant added in the mobile phase.

Consequently, each solute is involved in two different equilibria; the primary equilibrium occurs between the bulk aqueous mobile phase and the surfactant-modified stationary phase and the secondary between the aqueous phase and micellar aggregates. The latter corresponds to a further similarity with biological systems, which is the natural formation of micelles in extracellular and intracellular fluids by phospolipids, cholesterol, fatty acids, and triglycerides with proteins (lipoproteins) [137].

MLC can be considered as a more environmentally friendly technique compared to the traditional reversed-phase liquid chromatography because it operates with significantly reduced amounts of organic solvents or even none at all. Organic solvents, when used, are typically limited to small proportions, depending on the properties of the micelle and the organic modifier, typically up to 15–20%. Higher concentrations can disrupt and break down micelles [136]. This limitation constrains the ability to minimize analysis time and enhance method throughput under gradient conditions.

Retention on MLC is mainly governed by hydrophobic interactions, but the electronic and steric features of analytes can also influence their elution [138] Although different types of surfactants can be employed, the neutral polyoxyethylene (23) lauryl ether (Brij-35) is the most common surfactant used, in particular in ADMET properties investigation and ecotoxicological studies [137–145]. In this case, MLC is referred also as biopartitioning micellar chromatography (BMC). Next, the anionic sodium dodecyl sulphate (SDS) [144–147] and the cationic cetyltrimethylammonium bromide (CTAB) [145, 148] are often used, leading to a charged surface. Since the stationary phase surface is modified by adsorption of the selected surfactant, changing the surfactant also necessitates replacing the reversed-phase column.

MLC can be employed as a mode of HPLC for the separation of various compounds before their quantification using an appropriate detector. Ibrahim et al. used MLC for the separation and determination of betahistine (BHS) in the presence of its pharmacopeial impurity 2-(2-hydroxyethyl)pyridine (HEP) using a photodiode array detector [144]. Bhamdare et al. employed MLC with a photodiode array detector to separate and determine the insecticides monocrotofos (MCF), imidacloprid (ICP), dimethoate (DM), and profenofos (PFF) in spinach and chickpea leaves [149]. It is worth noting that MLC is considered as a green HPLC mode because micelles are an ecologically friendlier alternative to the organic modifiers commonly added to the mobile phase to facilitate the elution of hydrophobic analytes and the waste produced by MLC is non-toxic [144, 149]. However, MLC is mainly used in drug discovery and environmental sciences for the estimation of ecotoxicological endpoints. More to the point, MLC has been successfully employed for the prediction of blood–brain permeation of drugs [146, 150] or other compounds (e.g., saponins) [143], human oral absorption [137, 139, 141, 150–152], volume of distribution [138, 140, 147, 153], half-life [138, 153], clearance [138, 140, 153], plasma protein binding [140, 141, 147, 153], skin permeability [154], as well as therapeutic parameters (e.g., therapeutic dose, lethal dose to 50% of population, LD_50_) [140]. MLC has also been used for the assessment of pesticide safety, evaluating potential hazards to humans due to dermal absorption [147] and predicting ecotoxicological indices (e.g., LD_50_ or lethal concentration to 50% of population, LC_50_) to certain aquatic organisms as well as honey bees [145].

### Immobilized liposome chromatography

Liposomes are self-assembled structures that occur naturally but they can also be easily prepared in laboratory using acylphosphatidylcholines, cholesterol, and charged lipids (e.g., stearylamine, dipalmitoylphosphatidylglycerol, phosphatidylethanolamine) [155]. Their main characteristic is the presence of two lipid bilayers, in contrast to micelles, which contain only one [156]. Liposomes can be produced in a variety of sizes, membrane composition and layer structures using techniques, such as sonication, extrusion, and homogenization [155]. The most common phospholipids used to form liposomes include phosphatidylcholine, phospatidyl serine, phosphatidyl ethanolamine, phosphatidyl glycerol, and phosphatidic acid often in combination with cholesterol [156].

Liposomes serve as excellent biomimetic models for drug interaction studies and are utilized in both liquid chromatography and capillary electrophoresis, particularly for simulating the behavior of natural cell membranes [157–159]. In immobilized liposome chromatography (ILC), liposomes, formed from phosphatidylcholine or unilaminar phospholipids, are typically entrapped into agarose-based gels or silica particles through steric, hydrophobic, electrostatic, or covalent interactions [160]. The immobilized liposome stationary phases effectively mimic biomembrane system due to the lipid bilayer structure of liposomes and the enhanced fluidity of lipid molecules [158, 161]. The main application of ILC lies in the screening and analysis of membrane-permeable compounds [162]. The limitations of ILC include the instability of liposomes, the complexity involved in their preparation and immobilization onto solid supports, limited reproducibility and robustness, relatively low throughput due to the need for careful maintenance of liposome integrity and high costs associated with the large amounts of liposomes and samples required for the method [163].

ILC has been employed to separate free and encapsulated bioactive species, as well as to investigate interactions between solutes and phospholipids [155]. Zou et al. utilized ILC to screen and analyse permeable compounds in Radix Angelica Sinensis [161] and to separate compounds present in Danggui Buxue decoction (a combined Chinese prescription of Radix Astragli:Radix Angelica Sinensis (5:1) for treating all kinds of ischemia) that interact with the liposome membrane [160]. In this case, the stationary phase was prepared by dissolving phosphatidylcholine in chloroform, mixing the solution with silica and then evaporating the solvent. The resulting PC film-coated porous silica gel was swollen in a buffer, washed and packed into a column [160, 161]. Another notable application involves the isolation of proteins and the investigation of their potential separation through stimulus reaction in immobilized aqueous two-phase systems. Current separation methods leverage changes in protein conformation and the control of stimulating protein-membrane interactions for the separation of other proteins [158]. A modification of ILC was proposed by Hou et al., who developed receptor liposome biomembrane chromatography by immobilizing the receptor protein α-glucosidase in porous silica gel with liposome vesicles to screen and analyze permeable compounds in natural medicinal herbs [162].

## Liposome electrokinetic capillary chromatography

Electrokinetic capillary chromatography (EKC) belongs to electromigration techniques. The sample is introduced into a capillary filled with a buffer solution, containing a ligand, which may be biomimetic. The ligand interacts with the solutes, functioning as a pseudostationary phase [156]. Thus, the separation mechanism relies on the distribution of analytes between an aqueous phase and the freely moving pseudostationary phase, as well as the electrophoretic mobility of the analytes. A widely used biomimetic pseudostationary phases can be constructed using liposomes made from natural or synthetic phospholipids [163]. Liposomes can also be dynamically or covalently attached to fused-silica capillary for analysis using open-tubular capillary electrochromatography (OT-CEC) [163]. This approach allows the creation of a stable coating that can withstand a large number of injections of analytes [163].

Liposome electrokinetic capillary chromatography (LEKC) effectively mimics biological membranes and can be employed to study analyte-biomembrane interactions [159, 163] or lipid-water partition coefficients [164]. LEKC is also considered as a measure of lipophilicity [159]. Wang et al. predicted human oral absorption of 27 organic neutral compounds using egg phospholipid and soybean phosphatidylserine liposomes [165]. Amezqueta et al. postulated that electrokinetic chromatography using lecithin liposomes, particularly lecithin microemulsion as the pseudostationary phase can emulate skin partition of neutral solutes [157]. Orzel et al. postulated that liposome electrokinetic capillary chromatography is superior than IAM chromatography for simulating the partitioning process in the pulmonary delivery of drugs [166]. Limitations of LEKC include the instability of liposomes, the complexity of their preparation, the potential for hydrophobic analytes to bind irreversibly, the need for specialized equipment, the high cost of reagents, and the requirement for skilled personnel. A further limitation of LEKC is that it is not suitable for investigating of highly hydrophilic neutral and ionized compounds, resulting in narrower range of applicability concerning lipophilicity compared to IAM chromatography [166].

Recently, LEKC has been employed for chiral separations. The first application was demonstrated by the work of Li et al., who successfully separated the enantiomers of four racemic drugs [167]. In addition to liposomes, other biomimetic pseudostationary phases have been utilized in electrokinetic capillary chromatography for chiral separations. Specifically, Lanaro et al. proposed the use of human serum albumin as a pseudo-stationary phase in electrokinetic chromatography to achieve enantioseparation of R- and S-propranolol [168]. Bile salts, which are chiral surfactants, represent another biomimetic component that can be employed in electrokinetic chromatography for the separation of chiral drugs, amino acids, pesticides and phytochemicals [169].

## Separations using biomimetic magnetic nanoparticles

Magnetic nanoparticles are characterized by para-magnetism, large surface area, and high surface energy, making them highly suitable for biological separation and purification [170, 171]. Initially, the target species of interest attach to the magnetic carrier/nanoparticles, which are then separated under an external magnetic field. However, magnetic nanoparticles lack specificity, necessitating functionalization with enzymes, proteins, and other biological agents to mimic the natural binding sites of biomolecules to achieve an efficient capture and separation of the specific target [171].

Santana et al. proposed dextran-coated magnetic nanoparticles modified with a protein A mimetic ligand for lgG purification. Dextran, a neutral polysaccharide, was chosen to modify the magnetic particle surface due to its negligible level of nonspecific adsorption [172]. Goyal et al. prepared acrylamide-based biomimetic magnetic nanoparticles by co-polymerizing acrylamide and ethylene glycol dimethacrylate in the presence of S-naproxen on silica-coated Fe_3_O_4_ nanoparticles. The material was applied to separate S-naproxen from R-naproxen and other similar drugs, such as ibuprofen and ketoprofen [173].

Liu et al. synthesized a novel biomineralized covalent framework (BM-COF) material based on magnetic silk fibroin. Nanoparticles were deposited by in-situ mineralization after co-precipitation and COFs were prepared by in-situ self-assembly of a COF layer on the Fe_3_O_4_@silk fibroin surface using interfacial directional growth technology. The BM-COFs were successfully applied for the separation and enrichment of sulforaphane from cruciferous vegetables [174].

Wang et al. used amino group-modified magnetic nanoparticles as substrates and water-soluble self-polymerizable dopamine as the imprinting monomer to prepare myoglobin-imprinted magnetic nanoparticles. The affinity-determining factors involve hydrogen bonding, electrostatic interactions and physical matching of imprinting cavities. The material was successfully applied to human serum for the extraction of myoglobin for diagnosing diseases like acute myocardial infarction, acute myocardial ischemia, and muscle injury [175].

Another interesting approach is the preparation of biomimetic nanoparticles modified with naturally derived active cell membranes which results in nanoparticles with enhanced biointerfacing capabilities [176]. The functionalization should retain the complexity of the cell membrane in order the membrane-camouflaged nanoparticles to exhibit many of the properties of the source cell [177].

Rao et al. fused platelet and leukocyte membranes and coated the prepared hybrid membranes onto magnetic beads, subsequently modifying their surface was modified with specific antibodies. These biomimetic cell-membrane-camouflaged nanoparticles were successfully used for the efficient and specific isolation of circulating tumor cells (malignant cells shed by solid tumors into the circulatory system) [178].

Li et al. prepared biomimetic immuno-fluorescent magnetic multifunctional nanoprobes, consisting of magnetic γ-Fe_2_O_3_ and fluorescent quantum dots as the core, and leukocyte membrane vesicles with antibodies as the shell. These leukocyte membrane-coated fluorescent magnetic nanoparticles with antibodies (LFMNPs-Ab) can bind to three types of breast cancer cells with varying Her2 marker levels. These cells exhibit different magnetic susceptibilities when exposed to a constant external magnetic field, allowing for their magnetic separation and collection from blood samples and, thus, resulting in targeted separation of tumor cell subpopulations for diagnostic purposes [179].

Cell membranes can also express specific receptors and, therefore, the functionalization of magnetic nanoparticles with certain cell membranes receptors can result in a promising tool in the drug discovery process in order to target and separate bioactive compounds according to their affinity to a certain receptor. Hue et al. used dual functionalization of carbon nanotubes with magnetic nanoparticles and α_1A_-adrenergic receptor (α1Α) (member of the G protein-coupled receptor superfamily) HEK 293 cell membrane. The prepared platform exhibited high binding capacity, satisfactory selectivity, and rapid separation ability and was used for the separation of potential bioactive compounds, such as benzoylmesaconine and lappaconitine, from Traditional Chinese medicine extracts [177].

Zhou et al. immobilized membrane fragments from cells expressing SNAP-Tag-epidermal growth factor receptor (EGFR) on the surface of magnetic nanoparticles. They were utilized as a drug discovery platform in order to screen for the EGFR-targeting active compounds of Zanthoxyli Radix [171].

The broader application of biomimetic magnetic nanoparticles in separation science and technology necessitates addressing their drawbacks, such as their relatively high production costs, limited reusability, potential toxicity, and the need to enhance specificity. Additionally, challenges such as magnetic field limitations—particularly in large-scale operations or with highly viscous samples—environmental concerns regarding their disposal and stringent regulatory standards for drug and protein purification must be resolved [180].

## Biomimetic separation techniques: features, strengths, and limitations

Biomimetic separations exhibit distinct features, strengths, and limitations compared to traditional separation approaches, such as liquid–liquid extraction, solid-phase extraction, normal-phase, reversed-phase, size-exclusion, ion-exchange chromatography, as well as capillary electrophoresis. Traditional separation techniques predominantly rely on differences in physicochemical properties, including hydrophobicity/lipophilicity, polarity, molecular size and charge [181, 182] are cost-effective, versatile, and scalable for industrial applications [183, 184]. However, they often struggle with complex matrices or closely related species, leading to increased analysis time and thereupon solvent consumption. This provokes higher costs especially for large-scale applications [181, 182] and poses significant environmental concerns, although greener alternatives [185, 186] and procedures [187, 188] are emerging.

Biomimetic separation techniques rely on the specific recognition properties of specialized biomimetic (nano)materials and surfaces [4, 67–72, 189] and incorporate higher information content. These methods excel in targeting specific compounds with minimal interferences from other components present in the sample matrix, but involve higher initial costs for specialized stationary phases or reagents [12, 17, 18, 31, 32, 34, 40, 110, 111]. Despite the expense and potential environmental concerns from hazardous chemical use [173, 174], biomimetic materials can often be used for a long time with reduced solvent consumption, partially offsetting costs [4, 49, 52, 96]. Scalability remains a challenge, though advancements in synthesis are improving feasibility.

Both approaches can be time-intensive. Traditional methods may require multiple steps and/or lengthy conditioning, while biomimetic methods may need significant preconditioning of materials [4]. Biomimetic approaches with their high selectivity are particularly suited for biochemical species, enantiomers, and structurally similar compounds [4, 67–72, 84–86, 96, 104–106] as well as in modeling biological processes, thus providing twofold information, i.e., separation and permeability or distribution evaluation in living organisms. They are especially valuable to rapidly screening ADME properties of drug candidates and other bioactive compounds [10–14, 33, 34, 36, 37, 40, 43–45, 58] and for ranking chemicals according to their toxicity potential to humans [46] and ecosystems [31, 32, 47, 48].

A comparative overview of traditional and biomimetic separation techniques is presented in Table 3. The optimal choice depends on the specific requirements of the application, including the nature of the analytes, the complexity of the matrix as well as economic considerations.

**Table 3:** Comparison of traditional vs. biomimetic separation techniques in various relevant aspects

	Traditional separations	Biomimetic separations
Efficiency	High for general applications	High for specific targets
Selectivity	Moderate to high	Moderate to very high
Cost	Moderate initial cost- Higher consumption of consumables	Higher initial cost (specific reagents/ consumables)—Lower running cost
Time	Moderate to fast	Time-saving for specific targets
Scalability	High	More limitations
Environmental impact	Solvent- intensive	Potentially greener, generally require less amounts of solvents
Applications	Broad range	Specialized

## Future trends and perspectives

Future research directions in biomimetic strategies may focus on enhancing the stability of biomimetic ligands through techniques, such as the use of stabilizers, selecting more robust supports, or expanding covalent immobilization methods. Hybrid biomimetic materials are likely to be developed by combining biomimetic ligands with nanotechnology (e.g., gold nanoparticles, graphene) to improve selectivity and durability. Integration with microfluidics can lead to lab-on-a-chip devices that incorporate biomimetic separation, particularly for rapid, point-of-care applications.

Reducing the cost of biomimetic approaches may involve developing materials that require smaller amounts of biomimetic ligands, extending the lifespan of biomimetic materials and stationary phases, as well as improving their regeneration ability. The principles of Green Chemistry are expected to influence the field, by introducing environmentally friendly synthesis and immobilization methods for producing biomimetic materials. Applications may expand to various fields, including industrial separation processes and environmental technologies, such as pollutant removal (biomimetic remediation) and water purification. Scaling up biomimetic technology will depend on designing efficient separation units and developing synthetic analogues (e.g., molecularly imprinted polymers, synthetic peptides) to overcome the limitations and costs associated with biological materials.

Advancements in computing technology and artificial intelligence (AI) are expected to further drive progress in biomimetic separations. Machine learning techniques could play a significant role in modeling ligand-receptor interactions and designing novel ligand structures with enhanced specificity and stability. Decades of accumulated knowledge on affinity ligand development can benefit from AI’s processing power to identify potential binders that might otherwise remain undetected. Big data analytics can extract valuable information from databases and suggest molecules for experimental testing. Results from these tests can then be fed back into algorithms to predict new candidates for testing, enabling iterative optimization cycles [189].

AI may also assist in simulating large-scale separations to predict performance and troubleshoot potential issues, monitor adjust biomimetic separations in real time, and integrate robotic systems for high-throughput experiment, testing a wide range of biomimetic materials.

## Conclusions

The emulation of biological systems, mechanisms, and processes, and their adaptation to separation, isolation, and purification, has led to the development of various biomimetic ligands and binding agents. This has resulted in numerous separation schemes for the complex separation and purification of proteins, antibodies, nucleic acids, enzymes, drugs, and other bioactive compounds, as well as for studying drug-receptor interactions and screening chemicals’ permeability, absorption and distribution, toxicity, and predicting environmental risks. Typical drawbacks include the high cost of biomimetic reagents and stationary phases, the commercial unavailability of some chromatographic columns, ligand instability, labor-intensive procedures for the preparation of biomimetic nanomaterials and stationary phases, and limitations related to the compounds being investigated (e.g., their lipophilicity).

Continued growth is anticipated in the future with new applications in areas such as Food, Pharmaceutical and Environmental Analysis, Forensics, Biochemistry, Pharmacology and Toxicology as well as Drug Design. Further advances will require addressing key challenges, such as reducing the cost of binding agents and stationary phases, developing new biomimetic stationary phases, increasing the commercial availability of biomimetic chromatographic columns, improving stability (e.g., through new immobilization techniques), developing of microcolumns to minimize solvent consumption and standardizing chromatographic conditions. In this regard, combinatorial chemistry, molecular simulation, 3D printing and artificial intelligence may play more significant roles.

## Data Availability

No datasets were generated or analysed during the current study.
